# NanoLuc Bioluminescence-Driven Photodynamic Activation of Cholecystokinin 1 Receptor with Genetically-Encoded Protein Photosensitizer MiniSOG

**DOI:** 10.3390/ijms21113763

**Published:** 2020-05-26

**Authors:** Yuan Li, Zong Jie Cui

**Affiliations:** Institute of Cell Biology, Beijing Normal University, Beijing 100875, China; liyuanswkx@163.com

**Keywords:** miniSOG, CCK1 receptor, NanoLuc, ^1^O_2_, calcium oscillations

## Abstract

In contrast to reversible activation by agonist, cholecystokinin 1 receptor (CCK1R) is permanently activated by singlet oxygen generated in photodynamic action, with sulphonated aluminium phthalocyanine or genetically encoded mini singlet oxygen generator (miniSOG) as photosensitizer. In these works, a halogen light source was used to power photodynamic action. For possible in vivo application of photodynamic CCK1R physiology, bearing a cumbersome light-delivery device connected to an external light source by experimental animals might interfere with their behavior. Therefore, in the present work, the possibility of bioluminescence-driven miniSOG photodynamic CCK1R activation was examined, as monitored by Fura-2 calcium imaging. In parallel experiments, it was found that, after plasma membrane (PM)-localized expression of miniSOG_PM_ in AR4-2J cells, light irradiation with blue light-emitting diode (LED) (450 nm, 85 mW·cm^−2^, 1.5 min) induced persistent calcium oscillations that were blocked by CCK1R antagonist devazepide 2 nM. NanoLuc was expressed bicistronically with miniSOG_PM_ via an internal ribosome entry site (IRES) sequence (*pminiSOG_PM_-IRES-NanoLuc*). The resultant miniSOG_PM_-IRES-NanoLuc-AR4-2J cells were found to generate strong bioluminescence upon addition of NanoLuc substrate coelenterazine. Strikingly, coelenterazine 5 microM was found to trigger long-lasting calcium oscillations (a hallmark for permanent CCK1R activation) in perifused miniSOG_PM_-IRES-NanoLuc-AR4-2J cells. These data indicate that NanoLuc bioluminescence can drive miniSOG_PM_ photodynamic CCK1R activation, laying the foundation for its future in vivo applications.

## 1. Introduction

The rhodopsin-type or A class calcium-mobilizing G protein-coupled receptor (GPCR) cholecystokinin type 1 (CCK1 or CCK1R) [1,2] is widely expressed both in the central nervous system (CNS) [3,4,5,6] and in peripheral tissues [7,8,9,10,11,12]. CCK1R in the CNS regulates neurotransmitter release [13,14,15], anxiety [16,17], appetite [18], neurogenesis [19], brain development [6], learning, and memory formation [20]. Peripheral CCK1R is involved in pancreatic [21,22] and adrenocortical secretion [23], gallbladder contraction [21], inhibition of gastric emptying and gastric acid secretion [24], lower esophageal sphincter relaxation [25], and slowing of colonic motility [26].

Owing to the wide distribution and participation in important physiological functions, CCK1R and coupled signaling pathways are rather well characterized [27], including coupling to calcium signaling [1,2,28]. In contrast to reversible activation by agonists, one strikingly conspicuous feature of CCK1 receptor pharmacology is that CCK1R such as those present at the basal plasma membrane in rat pancreatic acini are permanently activated by the lowest lying excited state of molecular oxygen, the delta singlet oxygen (^1^O_2_) [29,30,31,32].

We have previously found that photodynamically generated ^1^O_2_ with chemical photosensitizers sulphonated aluminum phthalocyanine (SALPC) or gadolinum porphyrin-like macrocycle B (GdPLMB) permanently activated CCK1R endogenously expressed in rat pancreatic acinar cells [29,30,31] or ectopically expressed in human embryonic kidney epithelial cell HEK293 [33]. Emergence in recent years of genetically-encoded protein photosensitizers (GEPPs) such as KillerRed or mini singlet oxygen generator (miniSOG) prompted us to extend our earlier works with chemical photosensitizers to these new GEPPs. We have found that the GEPPs KillerRed or miniSOG specifically expressed at the plasma membrane in rat pancreatic acinar tumor cell AR4-2J were able after light irradiation with a halogen cold light source to photodynamically activate CCK1R permanently [33].

The miniSOG is the first GEPP known to specifically generate ^1^O_2_ (quantum yield Φ_Δ_ ≥ 0.03) [34,35,36]. MiniSOG variants and other flavin-binding ^1^O_2_-generating protein photosensitizers with varied ^1^O_2_ quantum yields (up to 0.61) have also emerged [32,37,38,39,40].

The availability of a large arsenal of GEPPs might immediately enable in vivo study of photodynamic CCK1R physiology or pharmacology in CCK1R-expressing cells in both the CNS and peripheral tissues. However, a major challenge for such works might be the possible interference from bearing a cumbersome light-delivery device connected to an external light source, of the intrinsic animal behavior or function under investigation. Therefore, in the present work, the possibility of bioluminescence-driven miniSOG photodynamic CCK1R activation was examined. In parallel experiments, it was found that light irradiation with a blue light-emitting diode (LED) of AR4-2J cells expressing plasma membrane (PM)-localized miniSOG (miniSOG_PM_) resulted in persistent calcium oscillations that were completely blocked by CCK1R antagonist devazepide. When miniSOG_PM_ was expressed bicistronically with a miniaturized luciferase, NanoLuc, by way of an internal ribosome entry site (IRES, to ensure separate protein expression of NanoLuc and miniSOG) sequence, the resultant miniSOG_PM_-IRES-NanoLuc-AR4-2J cells emitted strong bioluminescent light upon addition of NanoLuc substrate coelenterazine. Addition of coelenterazine at micromolar concentrations to perifused miniSOG_PM_-IRES-NanoLuc-AR4-2J cells was found to trigger persistent cytosolic calcium oscillations, which lasted long after wash-out of substrate coelenterazine. These data indicate that NanoLuc bioluminescence could drive miniSOG photodynamic CCK1R activation, laying the foundation for future in vivo applications.

## 2. Results

**Figure 1:**
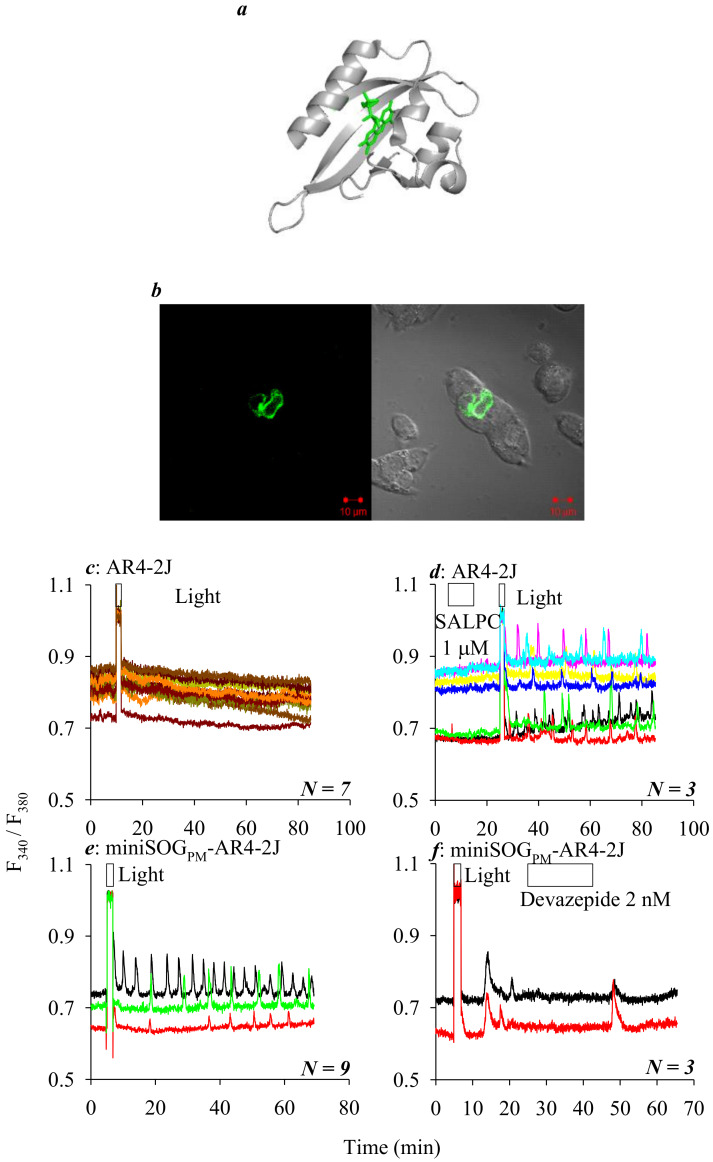
**Mini singlet oxygen generator (miniSOGPM) photodynamic activation of CCK1R in AR4-2J cells driven by a blue LED light source**. (**a**) Structure of miniSOG. The full amino acid sequence of miniSOG is from [37], and three-dimensional model built by Swiss-model. The miniSOG chromophore flavin mononucleotide (FMN) is outlined in green. (**b**) MiniSOGPM expression in AR4-2J, confocal images (λex 488 nm) were taken 24 h after transfection. Control non-transfected AR4-2J cells (**c**, *N* = 7; **d**, *N* = 3) or miniSOGPM-AR4-2J cells (**e**, *N* = 9; **f**, *N* = 3) loaded with Fura-2 AM were perifused, and sulphonated aluminum phthalocyanine (SALPC) 1 μM, devazepide 2 nM, red light (>580 nm, 36.7 mW·cm-2, 1.5 min) from a halogen cold light source (**d**), or blue light-emitting diode (LED) (450 nm, 85 mW·cm-2, 1.5 min) light (**c**,**e**,**f**) were applied as indicated. (**c**) Non-transfected AR4-2J cells with blue LED light irradiation. (**d**) Non-transfected AR4-2J cells exposed to SALPC 1 μM, followed by red light irradiation from halogen cold light source. (**e**,**f**) MiniSOGPM-AR4-2J cells with blue LED light irradiation. Note the complete inhibition of calcium oscillations by cholecystokinin 1 receptor (CCK1R) antagonist devazepide 2 nM (**f**, *N* = 3). Colored calcium traces tracings are shown with each from individual cells measured simultaneously. These original tracings shown are from 1 out of *N* (as indicated) identical experiments.

Fusion of a PM-localizing sequence (see Materials and Methods) to miniSOG (Figure 1a) obtained a PM-targeting miniSOG, miniSOG_PM_. After transduction of AR4-2J cells with plasmid *pminiSOG_PM_*, protein expression was confirmed by miniSOG_PM_ fluorescence microscopy (Figure 1b) and the effect of light irradiation was examined. In control non-transfected AR4-2J cells, cytosolic calcium concentration remained rather stable, and blue LED light irradiation (450 nm, 85 mW·cm^−2^, 1.5 min) induced no major effect (Figure 1c). In control non-transfected AR4-2J cells, perifusion with chemical photosensitizer SALPC (1 μM) in the dark had no effect; after wash-out of unbound SALPC, subsequent light irradiation with red light (λ > 580 nm, 36.7 mW·cm^−2^, 1.5 min) from a halogen cold light source triggered calcium oscillations that persisted long after cessation of light illumination (Figure 1d). Light irradiation with blue LED (450 nm, 85 mW·cm^−2^, 1.5 min) of miniSOG_PM_-AR4-2J cells triggered similar long-lasting or persistent calcium oscillations (Figure 1e), which were completely blocked reversibly by the CCK1R antagonist devazepide 2 nM (Figure 1f). These data together indicate that permanent miniSOG_PM_ photodynamic activation of CCK1R could be driven by blue LED (450 nm) light, to trigger persistent calcium oscillations in miniSOG_PM_-AR4-2J cells.

**Figure 2:**
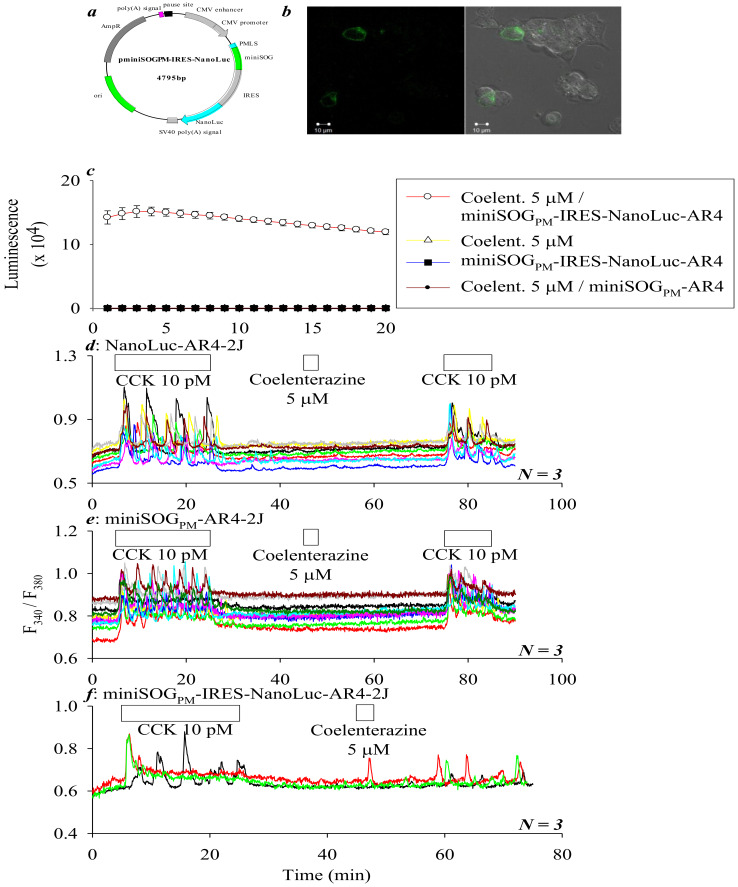
**MiniSOG_PM_ photodynamic CCK1R activation in AR4-2J cells driven by NanoLuc bioluminescence light.** (**a**) Plasmid *pminiSOG_PM_-IRES-NanoLuc*. PMLS: plasma membrane-localizing sequence. IRES: internal ribosome entry site. (**b**) MiniSOG_PM_-IRES-NanoLuc-AR4-2J cells, with confocal images (λ_ex_ 488 nm) taken 24 h after transfection with *pminiSOG_PM_-IRES-NanoLuc*. (**c**) Bioluminescene light emitted after addition of NanoLuc substrate coelenterazine 5 μM, no bioluminescence light was detected under control conditions (as indicated) (mean ± SEM, *n* = 3). (**d**–**f**) Fura-2-loaded NanoLuc-AR4-2J (**d**), miniSOG_PM_-AR4-2J (**e**), or miniSOG_PM_-IRES-NanoLuc-AR4-2J cells (**f**) were perifused, and CCK (10 pM) and coelenterazine 5 μM (3 min) were applied as indicated by the horizontal bars. Colored calcium tracings are each from individual cells measured simultaneously. These tracings are from 1 out of *N* identical experiments (**d**–**f**, *n* = 3).

In the above experiments, for miniSOG_PM_ photodynamic CCK1R activation to occur, an external light source, blue LED (450 nm), was applied at a power density of 85 mW·cm^−2^ for 1.5 min. For possible in vivo applications, it would be desirable if one could make use of an internal light source, namely, bioluminescence. Will bioluminescence generated by NanoLuc be strong enough to power miniSOG_PM_ photodynamic CCK1R activation? It was found that, when miniSOG and NanoLuc were co-expressed in a bicistronic vector in AR4-2J cells, both proteins retained their full function. The expressed NanoLuc could indeed generate enough bioluminescence to trigger miniSOG_PM_ photodynamic CCK1R activation, as shown below (Figure 2).

In expression plasmid *pminiSOG_PM_-IRES-NanoLuc*, an IRES sequence was inserted in between *miniSOG_PM_* and *NanoLuc* (Figure 2a). Confocal imaging confirmed the expression of miniSOG_PM_ (Figure 2b). NanoLuc expression (i.e., bioluminescence light emission) was readily detected after addition of substrate coelenterazine 5 μM in miniSOG_PM_-IRES-NanoLuc-AR4-2J cells, but no bioluminescence was detected at all in a buffered solution of coelenterazine 5 μM alone, in miniSOG_PM_-IRES-NanoLuc-AR4-2J cells without coelenterazine addition, or with coelenterazine 5 μM addition to miniSOG_PM_-AR4-2J cells not expressing NanoLuc (Figure 2c).

Tandem doses of CCK 10 pM induced reproducible calcium oscillations in NanoLuc-AR4-2J cells, and CCK-induced calcium oscillations disappeared immediately after wash-out of CCK; the addition of NanoLuc substrate coelenterazine 5 μΜ in between the two CCK doses had no effect (Figure 2d). Sequential CCK 10 pM also induced robust calcium oscillations in miniSOG_PM_-AR4-2J cells; the addition of coelenterazine 5 μΜ in between had no effect on baseline calcium concentration either (Figure 2e). CCK 10 pM similarly induced calcium oscillations in miniSOG_PM_-IRES-NanoLuc-AR4-2J cells, these CCK-induced calcium oscillation peaks disappeared completely as expected after wash-out of CCK; subsequent addition of coelenterazine 5 μΜ to these same cells induced calcium oscillations that were persistently present long after wash-out of the added coelenterazine (Figure 2f). These data indicate that, in the absence of an external light source, the addition of NanoLuc substrate coelenterazine 5 μΜ after simultaneous expression of NanoLuc and miniSOG_PM_ in the CCK1R-expressing AR4-2J cells by way of a bicistronic plasmid (pminiSOG_PM_-IRES-NanoLuc) provides an efficient means to permanently activate the endogenously expressed CCK1R.

## 3. Discussion

In the present work, the GEPP miniSOG was expressed at the plasma membrane in rat pancreatic acinar tumor cell AR4-2J, and light irradiation with a blue LED (450 nm) light source of the miniSOG_PM_-AR4-2J cells triggered long-lasting cytosolic calcium oscillations that were blocked completely by CCK1R antagonist devazepide. Therefore, miniSOG photodynamic action of CCK1R could be powered not only with a halogen cold light source as reported by us previously [33], but also with a wavelength-defined LED light source at 450 nm. Further, both miniSOG_PM_ and NanoLuc were expressed simultaneously in AR4-2J cells by transduction with a bicistronic plasmid *pminiSOG_PM_-IRES-NanoLuc* with the insertion between the gene sequences of *miniSOG* and *NanoLuc* of an internal ribosome entry site (IRES) sequence. The resultant miniSOG_PM_-IRES-NanoLuc-AR4-2J cells were found to emit strong NanoLuc bioluminescence light upon addition of NanoLuc substrate coelenterazine. The addition of coelenterazine to perifused miniSOG_PM_-IRES-NanoLuc-AR4-2J cells was found to trigger long-lasting cytosolic calcium oscillations that persisted long after wash-out of the added coelenterazine. These data together suggest that miniSOG photodynamic activation of CCK1R could be powered not only with a blue LED light source, but also with NanoLuc bioluminescence. The bicistronic plasmid *pminiSOG_PM_-IRES-NanoLuc* method could potentially be used to study photodynamic CCK1R physiology or pharmacology of CCK1R-expressing cells in vivo either in the CNS or in peripheral tissues.

CCK1R is unique among the dozen or so calcium-mobilizing A class GPCRs we have examined (not shown) in that it is activated permanently by ^1^O_2_ generated in a type II photodynamic action with SALPC, GdPLMB, KillerRed, or miniSOG as the photosensitizer, all powered by a halogen cold light source, with the full-spectrum white light directly for KillerRed and miniSOG, or with red light after filtering out shorter wavelengths by a long-pass filter (>580 nm) for SALPC and GdPLMB [29,30,31,33]. In the present work, we have extended our previous findings by powering miniSOG_PM_ photodynamic activation of CCK1R with a blue LED (450 nm) as an external light source, and with NanoLuc bioluminescence light after addition of NanoLuc substrate coelenterazine to miniSOG_PM_-NanoLuc-AR4-2J cells as an internal (within the AR4-2J cells) light source.

MiniSOG was targeted to plasma membrane in AR4-2J cells, to emit green fluorescence (500 nm) (Figure 1a,b). Light irradiation with blue LED (450 nm, 85 mW · cm^−2^, 1.5 min) had no effect in parental AR4-2J cells (Figure 1c), but triggered long-lasting calcium oscillations in miniSOG_PM_-AR4-2J cells (Figure 1e), which were similar to calcium oscillations induced in parallel experiments in untransfected AR4-2J cells by photodynamic action with chemical photosensitizer SALPC (1 μM), triggered by red light (>580 nm, 36.7 mW · cm^−2^, 1.5 min) from the halogen cold light source (Figure 1d). CCK1 receptor antagonist devazepide 2 nM completely blocked blue LED light irradiation-induced calcium oscillations in miniSOG_PM_-AR4-2J cells, unambiguously confirming blue LED-driven miniSOG_PM_ photodynamic activation of CCK1R (Figure 1f).

**Figure 3:**
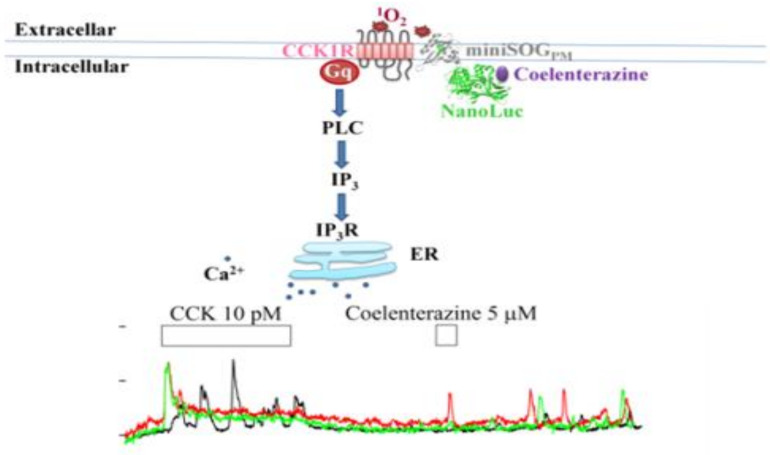
**NanoLuc bioluminescence-driven miniSOG_PM_ photodynamic CCK1R activation.** MiniSOG was expressed at the plasma membrane (PM), in a bicistronic *miniSOG_PM_-IRES-NanoLuc* construct, resulting in the expression of separate miniSOG_PM_ and NanoLuc proteins. The addition of NanoLuc substrate coelenterazine 5 μM to perifused miniSOG_PM_-IRES-NanoLuc-AR4-2J cells triggers the miniSOG_PM_ generation of singlet oxygen (^1^O_2_), likely via a radiationless NanoLuc to miniSOG_PM_ BRET process; ^1^O_2_ then oxidatively activates the endogenously expressed CCK1R in rat pancreatic acinar tumor cell AR4-2J. Gq, G protein q subtype; PLC, phospholipase C; IP3R, IP3 receptors; ER, endoplasmic reticulum.

NanoLuc (19 kDa), a miniaturized luciferase originally from deep sea shrimp *Oplophorus gracilirostris*, could use either coelenterazine or synthetic furimazine as substrate, to emit blue light peaking at 478 and 460 nm, respectively [41,42]. The emitted blue bioluminescence light is readily absorbed by miniSOG (λ_ex_ 448 nm) [34,35,36] (see text below and Table A1 for further details). NanoLuc has actually been used before quite widely to study protein–protein interactions [43], to regulate gene expression and cellular signaling [44,45], for molecular imaging [46,47], or for photodynamic killing of cancer cells [48,49]. The fusion construct NanoLuc–miniSOG with furimazine as substrate has been used to induce cancer cell killing in vitro [48,49,50,51]. It may be noted that, in these latter works, NanoLuc and miniSOG were expressed as single fused proteins NanoLuc–miniSOG- and NanoLuc–[GGGGS]-miniSOG [48,50]. Emission spectral studies confirmed NanoLuc–miniSOG bioluminescence resonance energy transfer (BRET) in expressing human breast adenocarcinoma cells SK-BR-3, because NanoLuc bioluminescence excitation of miniSOG fluorescence was readily detected. Further, NanoLuc bioluminescence was able to power miniSOG photodynamic SK-BR-3 cell killing [48,50]. In our own work with NanoLuc-IRES-miniSOG-AR4-2J cells, it is likely that NanoLuc and miniSOG were expressed as separate proteins. NanoLuc distribution or accumulation under the miniSOG-expressing PM would provide the platform for PM-delimited NanoLuc to miniSOG_PM_ BRET and therefore miniSOG_PM_ photodynamic CCK1R activation (Figure 3). This NanoLuc to miniSOG_PM_ BRET process probably needs to be further established, preferably in single cell microscopic spectral studies.

In the present work, simultaneous expression of miniSOG_PM_ and NanoLuc in the rat pancreatic acinar tumor cell AR4-2J was done with a bicistronic plasmid *pminiSOG_PM_-IRES-NanoLuc*. Addition of NanoLuc substrate coelenterazine 5 μM to miniSOG_PM_-IRES-NanoLuc-AR4-2J cells (with separate expression of miniSOG_PM_ and NanoLuc) led to strong bioluminescence light emission, which was not found in control experiments (coelenterazine alone; miniSOG_PM_-IRES-NanoLuc-AR4-2J cells without coelenterazine; coelenterazine added to miniSOG_PM_-AR4-2J cells) (Figure 2a–c). In separate experiments, coelenterazine addition at 5 μM to perifused miniSOG_PM_-IRES-NanoLuc-AR4-2J cells was found to trigger persistent and long-lasting calcium oscillations—a hallmark of miniSOG_PM_ photodynamic permanent activation of CCK1R (Figure 2f), but in NanoLuc-AR4-2J cells or miniSOG_PM_-AR4-2J cells, coelenterazine addition had no effect (Figure 2d,e). These data indicate very clearly that miniSOG_PM_ photodynamic activation of CCK1R could be powered with NanoLuc bioluminescence. One only needs to expose miniSOG_PM_-IRES-NanoLuc-AR4-2J cells to NanoLuc substrate coelenterazine at a relatively low concentration in the low μM range (Figure 3).

It may be noted that, for NanoLuc bioluminescence-driven miniSOG_PM_ photodynamic activation of CCK1R, NanoLuc substrates other than coelenterazine might also be used. In AR4-2J cells, however, the synthetic substrate furimazine may not be used, because low micromolar furimazine concentrations (such as at 5 μM) alone induced calcium increases in the absence of NanoLuc expression (data not shown).

It may be further noted that, although NanoLuc with coelenterazine emits at 478 nm maximally (100%), at 448 nm, the emission is still at 68% of the maximum [42]. MiniSOG is excited maximally at 448 nm (100%), but at 478 nm, the excitation spectrum is still at 77% of the maximum [35]. Therefore, there should be no problem for NanoLuc bioluminescence to excite miniSOG, for miniSOG photodynamic activation of CCK1R, which we observed as persistent cytosolic calcium oscillations (Figure 2f). Our work indicated that a match of NanoLuc maximal emission to 77% miniSOG excitation plus a match of 68% NanoLuc emission to miniSOG maximal excitation was sufficient (Table A1); an exact maximum-to-maximum or peak-to-peak match of the two is not necessary.

In conclusion, the recently emerged GEPP miniSOG could be used to photodynamically activate CCK1R permanently, not only with an external light source blue LED (450 nm) at a power density of 85 mWatt.cm^−2^ (1.5 min), but also with an internal light source in the form of simultaneously expressed NanoLuc upon the addition of NanoLuc substrate coelenterazine at 5 μM (3 min) (Figure 3) (see also Table A2, for a comparison of the parameters of these light sources). NanoLuc bioluminescence-powered permanent photodynamic CCK1R activation could be readily used for future in vivo applications without the need for cumbersome and complex optical delivery devices connected to an external light source, but with rather similar results in terms of permanent photodynamic CCK1R activation as with an external LED light source. This might lay the foundation for extensive studies of GEPP photodynamic CCK1R or other GPCR physiology or pharmacology.

## 4. Materials and Methods

### 4.1. Materials

Sulphated cholecystokinin octapeptide (CCK) and CCK1R antagonist devazepide were from Tocris Cookson (Bristol, UK). Dulbecco’s Modified Eagle’s Medium (DMEM) / F12 medium was bought from InVitrogen (Shanghai, China). Goods buffer 4-(2-hydroxyethyl)-1-piperazine-ethane-sulfonic acid (HEPES) was from Calbiochem (Darmstadt, Germany). Fura-2 AM was from AAT Bioquest (Sunnyvale, CA, USA). JetPRIME transfection reagent was from PolyPlus-transfection (New York, USA). Sulphonated aluminium phthalocyanine (SALPC) was from Frontier Scientific Inc. (AlPcS-834, Logan, UT, USA). Cell-Tak and Agar (Bacto^TM^) were from BD Biosciences (Bedford, MA, USA). Fetal bovine serum (FBS) was from Thermo Scientific (Shanghai, China). *pKillerRed_PM_* vector was bought from Evrogen (Moscow, Russia). *pNL1.1.CMV* (NanoLuc / CMV) vector containing the *NanoLuc* luciferase gene under the control of the cytomegalovirus (CMV) promoter and coelenterazine were from Promega (Madison, WI, USA). *PEF1A-IRES-Neo* vector was from Addgene (Watertown, MA, USA). Ampicillin and Kanamycin were from CWBio (Beijing, China). Endotoxin-free plasmid extraction kit and DH5à competent cells were from TianGen Biochemicals (Beijing, China).

### 4.2. AR4-2J and E. coli Cell Culture

AR4-2J was bought from ATCC (Rockville, MD, USA) and cultured in DMEM/F12 supplemented with 20% fetal bovine serum in a CO_2_ incubator under humidified atmosphere (5% CO_2_/95% air) at 37 °C, as reported before [1,2,28,33,52].

Solid *E. coli* medium LB/Kana and LB/Amp were sterilized and culture plates made. Liquid *E. coli* medium LB/Kana and LB/Amp had the same composition, but without agar.

### 4.3. Vector Constructs

A mammalian codon-optimized *miniSOG* gene (GenBank accession number JX999997) was synthesized de novo from nucleotides at Genscript (Nanjing, China) with the following full nucleotide sequence: ATGGAAAAGAGCTTTGTGATTACCGATCCGCGCCTGCCAGACAACCCGATCATTTTCGCGAGCGATGGCTTTCTGGAGTTAACCGAATATTCTCGTGAGGAAATTCTGGGTCGCAATGGCCGTTTCTTGCAGGGTCCGGAAACGGATCAAGCCACCGTGCAGAAAATCCGCGATGCGATTCGTGACCAACGCGAAATCACCGTTCAGCTGATTAACTATACGAAAAGCGGCAAGAAATTTTGGAACTTACTGCATCTGCAACCGATGCGCGATCAGAAAGGCGAATTGCAATATTTCATTGGTGTGCAGCTGGATGGCTAG. This synthesized full *miniSOG* gene sequence was inserted into plasmid *pKillerRed_PM_* (Evrogen, Moscow, Russia), to replace the *KillerRed* sequence. Competent *E. coli* were infected with the recombinant plasmid, cultured on solid LB/kana. Bacteria colonies were picked and further cultured in liquid LB/kana with shaking overnight. Proliferated plasmid was extracted for sequence verification. The obtained plasmid was named *pminiSOG_PM_* owing to the presence of a plasma membrane (PM) localization sequence in the original Evrogen plasmid. The transfected AR4-2J cells were named miniSOG_PM_-AR4-2J cells.

For construction of *pminiSOG_PM_-IRES-NanoLuc*, the coding sequence of *miniSOG_PM_* was cloned into plasmid *pNL1.1.CMV* (NanoLuc/CMV) (Promega) containing the *NanoLuc* luciferase gene under the control of the CMV promoter (Genscript, Nanjing, China). The *miniSOG_PM_* sequence was amplified from *pminiSOG_PM_* plasmid. The *IRES* sequence amplified from *PEF1A-IRES-Neo* vector was inserted between the two functional domains (NanoLuc and miniSOG_PM_). The plasmid construct was transformed into DH5a, harvested, and sequenced for verification. This plasmid was named *pminiSOG_PM_-IRES-NanoLuc*, and transduced AR4-2J cells were named miniSOG_PM_-IRES-NanoLuc-AR4-2J, where both miniSOG_PM_ and NanoLuc would express as separate proteins.

### 4.4. Transduction of AR4-2J Cells

AR4-2J cells were cultured in six-well plates or six-well plates with a round glass cover-slip in each well, and cells were allowed to grow to 50–70% confluence. Mixed plasmid (2 μg/well) and jetPRIME transfection reagent (4 μL/well) in jetPRIME buffer (200 μL) were added before the cells were cultured for a further 24 h. Transfection of AR4-2J cells was verified by NanoLuc bioluminescence (Tecan Infinite F200 Pro multimode reader) or miniSOG fluorescence confocal imaging (Zeiss LSM510 META, objective lens oil ×60).

### 4.5. miniSOG Fluorescence Imaging

Parental AR4-2J cells were planted on glass cover-slips and cultured overnight before transfection and further cultivation. At 24 h after transfection, cells were imaged in a confocal microscope (Zeiss LSM 510 META), under oil objective 63×/1.40, with λ_ex_ for miniSOG at 488 nm.

### 4.6. Photodynamic Action

To trigger photodynamic action, SALPC-bound cells were irradiated with red light (>580 nm, 36.7 mW·cm^−2^, 1.5 min) from a halogen cold light source (MegaLight 100, Hoya-Schott, Japan) equipped with condenser HLL201 and filter R60 (>80 nm).

MiniSOG_PM_-AR4-2J cells were irradiated with a blue LED (450 nm, 85 mW·cm^−2^, 1.5 min) (LAMPLIC, Shenzhen, China). Light irradiance at the level of attached cells in the Sykes–Moore perfusion chamber was measured with a power meter (IL1700, International Light Inc., Newburyport, MA, USA).

For NanoLuc bioluminescene to power miniSOG_PM_ photodynamic action, NanoLuc bioluminescence was first characterized and recorded in a Tecan Infinite F200 Pro multimode reader after addition of NanoLuc substrate coelenterazine. Measurements were carried out on living cells in a balanced buffer in 96-well plates with white walls (in triplicates). NanoLuc substrate coelenterazine (Promega) was added at a final concentration of 5 μM. Luminescence was recorded once every min. Coelenterazine 5 μM was added to perifused miniSOG_PM_-IRES-NanoLuc-AR4-2J cells (3 min) to trigger NanoLuc bioluminescence-powered miniSOG_PM_ photodynamic CCK1R activation.

### 4.7. Calcium Measurements

Transfected AR4-2J cells grown on glass cover-slips in six-well plates were loaded with Fura-2 AM (final concentration 10 μM, 1 h) directly after assembly in Sykes–Moore perfusion chambers. Cytosolic calcium was measured in an inverted fluorescent microscope (Nikon TE-2000U) coupled to a PTI (Photon Technology International Inc., now HORIBA, Edison, NJ, USA) calcium measurement system with alternating excitations of the loaded Fura-2 at 340 nm/380 nm (DeltaRam X), and emitted Fura-2 fluorescence (dichroic mirror 400DCLP, emitter 510 ± 40 nm) was detected with a charge-coupled device (CCD) camera (NEO-5.5-CL-3, Andor/Oxford Instruments, UK). Calcium concentration was expressed as F_340_/F_380_ ratios and plotted against time with SigmaPlot, as reported before [1,2,28,31,33,52,53]. In the figures shown in Results, the original colored calcium tracings were each from individual cells and only representative tracings from 1 out of *N* (as indicated, *N* ≥ 3) identical experiments were presented.

### 4.8. Data Presentation and Statistical Analysis

All data were presented as mean ± SEM. Student’s T-test was used for statistical analysis against controls and *p* < 0.05 was taken as statistically significant, as indicated (*). For calculation and comparison of induced calcium responses, calcium peak area above the baseline was integrated (usually per 10 min unless stated otherwise). All calcium tracings (each line tracing corresponds to an individual cell) and other data graphs were plotted with SigmaPlot.

## Abbreviations

CCK1R	Cholecystokinin 1 receptors
CNS	Central nervous system
GdPLMB	Gadolinium porphyrin-like macrocycle B
GEPP	Genetically-encoded protein photosensitizer
GPCR	G protein-coupled receptor
IRES	Internal ribosome entry site
miniSOG	mini singlet oxygen generator
LED	Light-emitting diode
^1^O_2_	Singlet oxygen
PM	Plasma membrane
SALPC	Sulphonated aluminium phthlaocyanine

## Aix A

**Table A1:** Comparison of NanoLuc emission and mini singlet oxygen generator (miniSOG) excitation parameters.

NanoLuc [42]			miniSOG [35]		
Peak	478 nm	100%		478 nm	77%
	448 nm	68%	Peak	448 nm	100%

These parameters were derived from figures in [35,42].

**Table A2:** Light sources to power miniSOG_PM_ photodynamic cholecystokinin 1 receptor (CCK1R) activation. LED, light-emitting diode.

Light Sources	Power Density	Duration (min)	References
Halogen cold light (white)	87 mW · cm^−2^	5	[33]
Blue LED (450 nm)	85 mW · cm^−2^	1.5	This work
NanoLuc + coelenterazine	5 μM (coelenterazine)	3	This work
